# Bibliographical

**Published:** 1897-11

**Authors:** 


### Bibliographical.

A Manual of the Injuries and Surgical Diseases of the Face, Mouth and Jaws. By John Sayer Marshall, M. D., (Syracuse University), former Professor of Dental Pathology and Oral Surgery, and Emeritus Professor of Oral Surgery of the Dental Department of Northwestern University, former Professor of Oral Surgery of the American College of Dental Surgery, etc., etc., etc. Published by the S. S. White Dental Manufacturing Company: Philadelphia.

In the preparation of this work the author has had in mind a change in the mode of teaching, deviating somewhat from the old mode of teaching by didactic lectures, and introducing the recitation system.

In the preface is the following statement: "During the past years the author has been more and more impressed with the disadvantages under which teachers and students have labored in the old system of teaching by didactic lectures. The same feeling has been growing year by year, among many of the teachers in the American medical and dental colleges, and many of them have expressed themselves as anxious to adopt a recitation system of teaching in their special departments. The author has endeavored to fill this requirement in the department of oral surgery, by the preparation of this volume. * * * In part first will be found those subjects which belong to the general principles of surgery, while part second is devoted to the more common injuries and surgical diseases which are associated with the face, the mouth and the jaws. These subjects have been divided into short chapters, suitable to class recitation work, and each chapter is followed by a series of review questions, covering the most important facts presented upon each topic. These questions can be used by the teachers as a basis for quizzes; they will also enable the student to quiz himself upon every subject presented."

This volume is an important addition to the textual literature of the dental profession, and will be greatly helpful, not only to the dental teacher, but to the medical teacher as well, and particularly those in the line of general surgery, especially when operations upon the jaws and face are to be performed. The work is divided into two parts, the first containing sixteen chapters and the second into forty-six. To each chapter there is appended a list of review questions that call attention to every important thought that has been presented in the text, and in such a manner as to well impress each particular on the mind of the student.

The author's equipment for the preparation of this work is of a high order, for in addition to a thorough medical education he has for a number of years been teaching surgery, and has had a large amount of surgical experience in both private and hospital practice. No special review of the work is here attempted. Upon many of the subjects presented there will be a variety of views entertained, this is always so, and as a matter of necessity, arising from difference of attainment and from views taken from different standpoints, but we are fully persuaded that this book will be highly appreciated by all interested in the subject. The volume should be in every student's hands, in every dental college library, as well as in the hands of every practitioner.

